# CRISPR-Editing of Sweet Basil (*Ocimum basilicum* L.) Homoserine Kinase Gene for Improved Downy Mildew Disease Resistance

**DOI:** 10.3389/fgeed.2021.629769

**Published:** 2021-05-12

**Authors:** Xiaoyu Zhang, Yee Chen Low, Michael A. Lawton, James E. Simon, Rong Di

**Affiliations:** Department of Plant Biology, Rutgers, The State University of New Jersey, New Brunswick, NJ, United States

**Keywords:** CRISPR, Cas9, gene editing, sweet basil, *Ocimum basilicum* L., homoserine kinase, downy mildew, *Peronospora belbahrii*

## Abstract

Sweet basil (*Ocimum basilicum* L.) downy mildew disease (DM) caused by *Peronospora belbahrii* is a worldwide threat to the basil industry due to the lack of natural genetic resistance in sweet basil germplasm collections. In this study, we used CRISPR-gene editing to modify the sweet basil DM susceptibility gene *homoserine kinase* (*ObHSK*). Gene-edited plants challenged with *P. belbahrii* displayed a significantly reduced susceptibility to DM, based on phenotypic disease indices and on *in planta* pathogen load. These results suggest that *ObHSK* plays a role in conditioning DM susceptibility, similar to that observed for the *AtHSK* gene in Arabidopsis. These results demonstrate the utility of CRISPR-gene editing in enhancing DM resistance and contributing to sweet basil breeding programs.

## Introduction

Sweet basil *(Ocimum basilicum* L.) is an important culinary herb and source of aromatic essential oils. Of all the *Ocimum* species, *O. basilicum* is the most susceptible to downy mildew (DM) disease, which is caused by the obligate Oomycete pathogen *Peronospora belbahrii* (Wyenandt et al., 2010; Homa et al., 2016). *P. belbahrii*, first reported in Uganda in the 1930s, was distinguished from other *Peronospora* species in 2005, and has become a global problem wherever sweet basil is grown (Belbahri et al., 2005; Thines et al., 2009; Wyenandt et al., 2010; Cohen et al., 2017). The pathogen produces chlorotic lesions on basil leaves with dark brown sporangia on the abaxial side of the leaf surfaces, greatly reducing basil quality and market value (Cohen et al., 2017). A substantial breeding effort has been made to identify and introgress DM resistance into commercial cultivars while simultaneously maintaining other key traits, such as aroma and taste (Wyenandt et al., 2015; Pyne et al., 2017, 2018). To-date, almost all sweet basil cultivars are DM susceptible, whereas resistant lines that incorporate genetic resistance from other *Ocimum* species exhibit sexual incompatibility with sweet basil (Cohen et al., 2017). At the same time, extant DM tolerant commercial cultivars are under constant threat from newly emerged *P. belbahrii* races (Wyenandt et al., 2018).

In this study, we used CRISPR/Cas (Clustered Regularly Interspaced Short Palindromic Repeats/CRISPR-associated)-gene editing technology to modify the sweet basil *ObHSK* gene, which encodes a homoserine kinase (HSK, EC 2.7.1.39). HSK belongs to the GHMP kinase family, catalyzes the reaction of L-homoserine (Hse) + ATP → O-phospho-L- homoserine (HserP) + ADP, which is an important step in the methionine and threonine biosynthesis pathway (Krishna et al., 2001). The structure and functions of HSK have been studied in bacteria and yeast (Zhou et al., 2000). In Arabidopsis, it has been identified as a DM recessive resistance factor and later its null mutation was reported to enhance the resistance to two strains of *Fusarium* (Van Damme et al., 2005, 2009; Brewer et al., 2014). CRISPR/Cas9, which cuts DNA at specific RNA-guided targets, can be used to modify or inactivate a gene. This technology has been rapidly adopted and widely used for genome engineering in various organisms and has been applied to plant breeding for disease resistance (Arora and Narula, 2017; Jiang and Doudna, 2017; Chen et al., 2019). The tetraploid nature of sweet basil, resulting from alloploidy (2n = 4x = 48) poses additional challenges to improvement via gene editing and breeding (Pyne et al., 2018).

Downy mildews are obligate biotrophic pathogens that penetrate into host tissues, grow intercellularly and form haustoria in mesophyll and epidermal cells for nutrient uptake (Van Damme et al., 2008). Both active defense mechanisms as well as the absence of host compatibility factors can result in plant resistance (Van Damme et al., 2005). Studies on the model plant *Arabidopsis thaliana* have identified several *downy mildew resistant* (*dmr*) mutants. *AtHSK* (*dmr1*) has been identified as a susceptibility gene that helps promote infection and colonization by the pathogen *Hyaloperonospora parasitic* (Van Damme et al., 2005, 2008, 2009). In this study, we used CRISPR to mutate the orthologous *ObHSK* gene to produce transgene-free, *ObHSK* gene-edited sweet basil plants. These results show that *ObHSK* mutant plants are resistant to *P. belbahrii*, compared to wild type sweet basil plants and these disease resistant gene edited plants grew normally in the greenhouse.

## Materials and Methods

### Construction of SB22 Sweet Basil *ObHSK* CRISPR-Editing Vector

**Figure 1:**
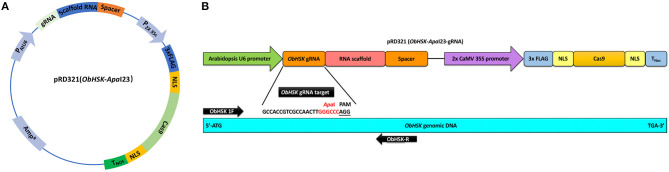
**(A)** Transient *ObHSK* CRISPR-editing vector pRD321. Expression of *ObHSK*-*Apa*I23 gRNA and the scaffold RNA is driven by the Arabidopsis U6 promoter. The humanized Cas9 (hCas9) gene with 3X FLAG and nuclear localization signal (NLS) is under the control of the 2X CaMV 35S promoter and the nopaline synthase terminator (T_Nos_). **(B)** The location of the *ObHSK*-*Apa*I23 gRNA target within the 5′ region of the *ObHSK* gDNA is indicated, together with the PCR primer set used to amplify the region containing and flanking the target site for mutant analysis.

The sweet basil *ObHSK* gene present in Rutgers breeding line SB22 was identified from RNAseq data (James E. Simon, unpublished data) using the NCBI tblastn tool with the *Arabidopsis thaliana* homolog gene *AtHSK/dmr1* (TAIR: At2g17265) as the query sequence. *ObHSK* cDNA and genomic DNA (gDNA) sequences were PCR-amplified using primers ObHSK-gDNA-F and ObHSK-gDNA-R (Supplementary Table 1) and the resulting PCR products sequenced. A 23 nucleotide (nt) guide RNA (gRNA) target sequence (5′-GCCACCGTCGCCAACTT*GGGCCC*AGG-3′) within the *ObHSK* gene from nucleotides (nt) #202–204 was selected according to the guidelines from Addgene (https://www.addgene.org/guides/crispr/). The selected gRNA target sequence starts with the “G” for the initiation of U6 promoter and includes an *Apa*I restriction sequence (*GGGCCC*), adjacent to the A/TGG PAM site, to facilitate mutant screening by restriction fragment length polymorphism (RFLP). The transient expression vector, pRD321, contains the *ObHSK*-*Apa*I gRNA fused to the 80-base scaffold RNA driven by the Arabidopsis U6 promoter as well as the dicotyledonous codon-optimized (using *Nicotiana* codon table) Cas9 gene driven by the 2 × CaMV 35S promoter (Figure 1A). After transformation into competent DH5α *E. coli* cells, the construct was purified using the PureLink^®^ Quick Plasmid Miniprep Kit (Invitrogen, Carlsbad, CA, USA) prior to biolistic delivery into sweet basil cells.

### Genetic Transformation of SB22 Sweet Basil Using Biolistic Bombardment

Mature seeds of *O. basilicum* breeding line Rutgers SB22 were surface sterilized with a solution of 30% bleach for 30 min followed by three 5 min rinses in sterile distilled water. Embryos were excised from seeds under a dissecting microscope and were sterilized with 10% bleach for 10 min and rinsed with sterile water. These surface sterilized embryos were then used directly for the biolistic transformation. The PDS-1000/He Particle Delivery System (Bio-Rad Laboratories, Hercules, CA, USA) was used to deliver pRD321 into freshly dissected basil embryos. Approximately 5 μg of pRD321 plasmid DNA was mixed with 2 mg of sterile gold particles (0.6 μm in diameter) suspension in 15 μL of 10 mM Tris (pH 8.0), 150 mM NaCl and vortexed for 10 s. Sixty microliters of DNA binding buffer [0.1 M spermidine, 25% PEG (1,300–1,600 MW) and 2.5 M CaCl_2)_] were added to the gold particle/plasmid DNA mixture and vortexed for 10 s, followed by a 10 min incubation at room temperature. DNA/gold particles were pelleted, resuspended in 70% ethanol and distributed onto four microcarrier membranes. Basil embryos were bombarded at 1,100 psi from a firing distance of 50 mm and then transferred to sweet basil callus induction medium [Murashige and Skoog (MS) salts, 3% (w/v) sucrose, 0.3% (w/v) Gelzan^TM^, 0.4 mg/L 6-benzylami-nopurine (BAP), 0.4 mg/L naphthalene acetic acid (NAA), pH 5.8] and cultured for 4–6 weeks in the dark. Induced somatic embryogenic calli were then transferred to sweet basil regeneration medium [MS salts, 3% (w/v) sucrose, 0.3% (w/v) Gelzan^TM^, 2 mg/L BAP, pH 5.8] for shoot induction. Since pRD321 is a transient vector and lacks an antibiotic resistance gene for selection of transformants in plants, antibiotics were omitted from the media. Shoot induction plates were placed under 16/8 h light/dark photoperiod at 22°C for 4–6 weeks. Each regenerated shoot was transferred to rooting medium [MS salts, 3% (w/v) sucrose, 0.3% (w/v) Gelzan^TM^, 1 mg/L IBA, pH 5.8] for root regeneration, prior to transfer to soil.

### *P. belbahrii* Inoculation on SB22 Sweet Basil Plants for Qualitative and Quantitative Assessment of DM Disease Progression and Severity

*P. belbahrii* sporangia spores were prepared by agitating freshly sporulating leaves of infected plants in distilled water for 30 min, filtered through a 40 μm nylon mesh cell using 50 mL of distilled water, counted in a hemocytometer and adjusted to 5 × 10^4^ spores mL^−1^. The spore solution was spray-inoculated onto plants using a Preval pressure sprayer (Wyenandt et al., 2015). Inoculated plants were incubated in a dew chamber for 2 days, which was maintained at 100% relative humidity and leaf wetness using Trion 707U series atomizing humidifiers (Trion, Sanford, NC, USA). Samples from the third leaf counting from the top of each inoculated plant were collected 5 days after inoculation. Qualitative assessment of disease progression was performed visually using an established disease severity index (Wyenandt et al., 2015) to compare symptoms of WT, T_0_ and T_1_ *ObHSK*-edited plants. Pictures were taken 15 days post-inoculation (dpi). Quantitative analysis of *P. belbahrii* levels *in planta* was performed by qPCR using β-tubulin as the plant endogenous gene and the ITS2 gene sequence for the pathogen. Primers (Supplementary Table 1) for each gene were designed using the Primer Express software (Applied Biosystems, Foster City, CA, USA). All samples were analyzed by SYBR green qPCR which was run on the default setting at 95°C for 3 min for the initial denaturation and 40 cycles at 95°C for 30 s followed by 60°C for 30 s with the StepOne instrument (Applied Biosystems, Foster City, CA, USA). The fold-change of gene expression was calculated by the 2^∧^^−ΔΔCt^ method and the qPCR analysis was performed in triplicate for each sample using three different leaf samples (Di et al., 2010).

### gDNA Extraction, PCR-Sequencing, and CRISPR-Editing Data Analysis

gDNAs of all samples were extracted using the GenElute^™^ Plant Genomic DNA Miniprep Kit (Sigma-Aldrich, St. Louis, MO, USA). The DNA concentration for each sample was measured with a NanoDrop/2000 spectrophotometer (Thermo Fisher, Waltham, MA, USA). All T_0_ and T_1_ plants were verified for the presence of the transgene (plasmid sequence of pRD321) using the primers of RD321-F and RD321-R that flank the sequence between the AtU6 promoter and the 35S promoter. The 321 bp gDNA fragment flanking the gRNA target site was amplified using the primer pair ObHSK-F and ObHSK-R. PCR products were purified using the GeneJET Gel Extraction Kit (Thermo Fisher, Waltham, MA, USA), prior to Sanger sequencing by Genewiz, Inc. The DNA sequencing chromatogram data for each T_0_ and T_1_ sample was analyzed using the Inference of CRISPR Editing (ICE) software tool (Synthego Performance Analysis, ICE Analysis. 2019. v2.0. Synthego) to predict the nature and efficiency of gene editing events.

## Results

### Identification of *DMR1* (*HSK*) Ortholog Gene for SB22 Sweet Basil

Transcriptomic data of DM-susceptible Rutgers SB22 sweet basil were obtained by RNAseq analysis (unpublished). *ObHSK* was identified using tblastn (NCBI) to query the transcriptome of SB22 with the *AtHSK*/*DMR1* amino acid sequence (GenBank accession # NP_179318.1). The alignment between *AtHSK* and *ObHSK* amino acid sequences showed 79.20% similarity. The complete gDNA sequence of SB22 *ObHSK* was PCR-amplified, cloned and sequenced. *ObHSK*, like *AtHSK*, lacks introns. The conserved region of *ObHSK*, comprising nt #181–1, 125, belongs to the superfamily cl30330. The sequence alignment among seven individual clones of *ObHSK* showed seven different alleles (Supplementary Figure 1A), with wt10 being identical to that of Genoveser (Navet and Tian, 2020) and to sequences obtained from our RNAseq analysis. The amino acid sequence alignment (Supplementary Figure 1B) among these seven clones showed ~7.5% divergence.

### Generation of SB22 Sweet Basil *ObHSK*-Edited Mutants by CRISPR-Gene Editing

A 23 nt gRNA editing target sequence, termed *ObHSK-Apa*I, is comprised of nt #202–204 of *ObHSK* (Figure 1B), which is conserved among seven clones sequenced (Supplementary Figure 1B). It contains an *Apa*I restriction sequence (*GGGCCC*) immediately upstream of the PAM site, which is either AGG or TGG (5′-GCCACCGTCGCCAACTT*GGGCCC*A/TGG-3′) (Supplementary Figure 1B). The pRD321 transient CRISPR-editing vector was constructed to transiently express the fused *ObHSK* gRNA and the scaffold RNA from the Arabidopsis U6 promoter and the codon-optimized humanized *Streptococcus pyogenes* Cas9 gene driven by the CaMV 2 × 35S promoter (Figure 1A).

**Figure 2:**
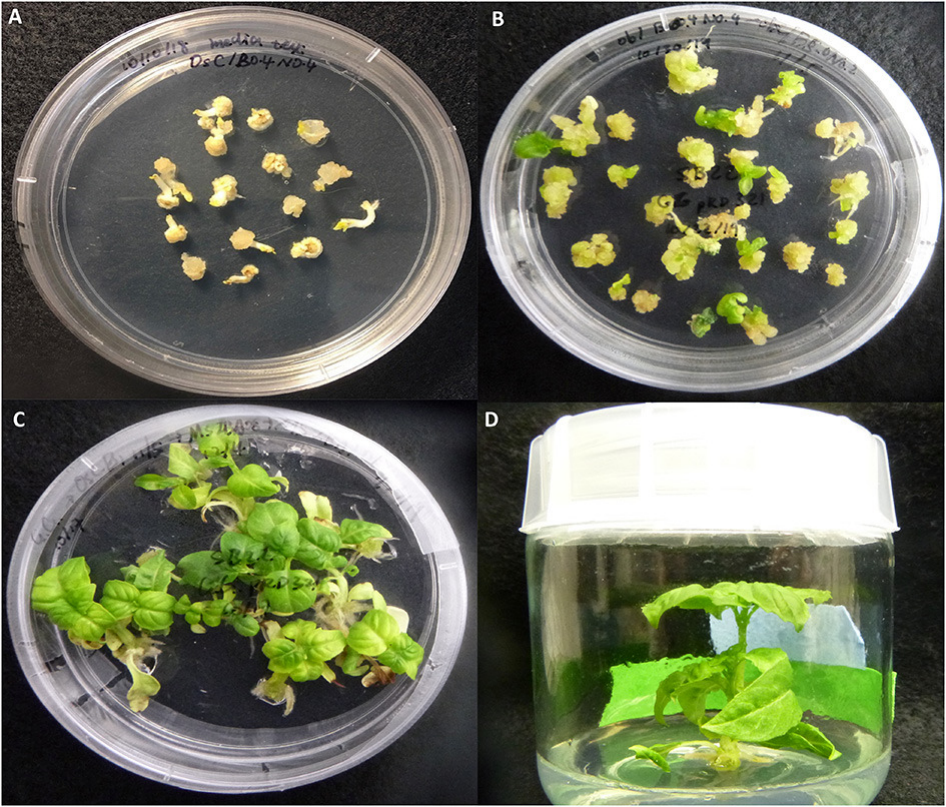
Transformation and regeneration of SB22 following biolistic bombardment with the transient CRISPR-editing vector pRD321. **(A)** Callus induced from bombarded embryos, **(B)** Shoot induction from calli, **(C)** Shoot culturing and elongation, and **(D)** Root induction.

Genetic transformation of SB22 sweet basil with pRD321 was performed by biolistic bombardment on embryo explants derived from mature seeds. One hundred bombarded mature embryos were placed on callus induction medium for 2 weeks to induce somatic embryogenic calli (Figure 2A). Calli developed from 88 out of the 100 bombarded embryos. Calli from individual embryos were separately transferred to shoot regeneration medium (Figure 2B). Shoots were regenerated within 4–6 weeks, with some embryos producing multiple shoots (Figure 2C). Shoots emerging from callus clusters derived from single embryos were individually transferred to root induction medium (Figure 2D). A total of 22 T_0_ plantlets were regenerated from 15 embryos and subsequently transferred to soil in the greenhouse.

### Molecular Characterization of *ObHSK*-Edited SB22 Sweet Basil Mutants

Regenerated plantlets were assessed for loss of the *Apa*I site at the CRISPR target site. This RFLP analysis attempted to identify mutants among the 22 regenerated plants based on *Apa*I digestion of the 321 bp gDNA fragment amplified by PCR with primers ObHSK-F and ObHSK-R (Supplementary Table 1). Cloning and sequencing of cumulative undigested PCR fragments from eight T_0_ plants revealed only one mutated sequence each from plants 321-5 and 321-13. This low efficiency may reflect the polyploid nature of the sweet basil genome, a problem that may be aggravated by incomplete *Apa*I digestion of PCR fragments. To efficiently identifymutations in these plants, Synthego online ICE analysis was adopted as described below.

Opportunistic integration of transient CRISPR-gene editing vectors into the plant genome can lead to additional editing in subsequent generations (Zhang et al., 2016). Consequently, genomes of T_0_ regenerated plantlets were first verified by PCR using pRD321-specific primers RD321-F and RD321-R (Supplementary Table 1) for the presence or absence of plasmid sequences. Among the eight T_0_ plants that produced putative *Apa*I undigested fragments in the RFLP analysis, lines 321-4, −7, and −8 were confirmed to have transgenes present while the remaining lines (321-5, 10, 12, 13, 14) were transgene-free (Supplementary Figure 2).

**Figure 3:**
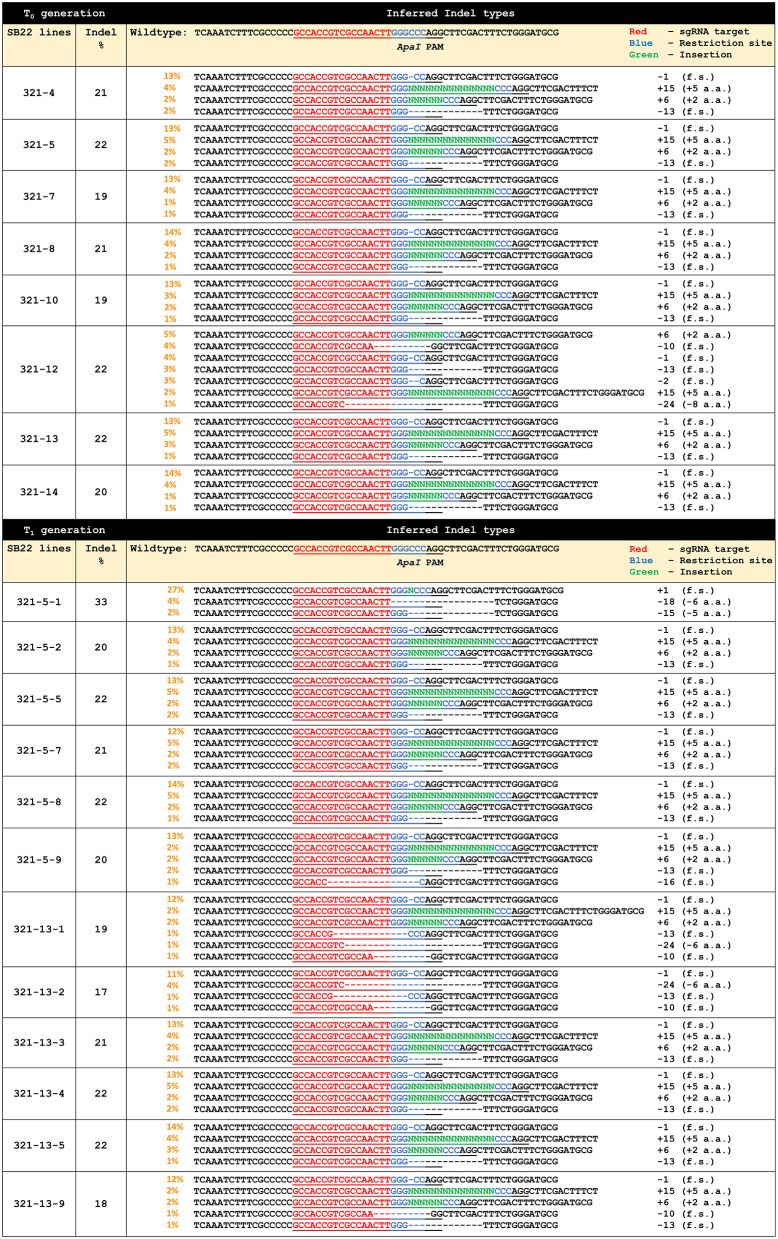
Gene editing profiles generated by ICE analysis for individual T_0_ and T_1_ *ObHSK*-edited sweet basil plants. “+” indicates addition of nucleotide base (N). “–” indicates deletion of nucleotide base. “f.s.” indicates frameshifting mutations. “PAM” (AGG) indicates the protospacer adjacent motif for Cas9. “a.a.” stands for amino acid.

*ObHSK-Apa*I target sequences in each T_0_ plant were genotyped and analyzed via the PCR-Sanger sequencing workflow. DNA sequencing chromatograms generated for each plant were analyzed using the Synthego ICE software to infer Indel types in putative *ObHSK* mutants, compared to the WT plant. Figure 3 shows the inferred CRISPR editing data for all T_0_ generation lines. Lines 321-4, 5, 7, 8, 10, 13, and 14 have four different types of editing patterns with respective Indel frequencies at 21, 22, 19, 21, 19, 22, and 20%. The inferred Indel types include two deletion events (-1 and −13 nt) which result in frameshifting mutations and two insertion events (+6 and +15 nt) which result in amino acid additions. Line 321-12 was predicted to also carry the −1, −13, +6, and +15 nt edited types, as well as three additional deletion types at −2, −10, and −24 nt, representing 22% of all Indels. Deletions of 2 and 10 nt are frameshifting mutations while deletion of 24 nt results in the net loss of 8 amino acids. Lines 321-5 and 321-13 were chosen for propagation to the T_1_ generation, due to their having the highest frequency (22%) of Indels and the absence of pRD321 sequences from the genome. Line 321-12 also has 22% Indels and displays a more diverse editing pattern, but was not chosen for T_1_ segregation because of growth retardation and sterility observed in the T_0_ generation.

Thirty seeds derived from each of the T_0_ lines 321-5 and 321-13 were germinated and propagated and these T_1_ plants were inoculated with *P. belbahrii* to assess their DM resistance. Thirty WT plants were also inoculated as controls. Symptoms on some 321-5 and 321-13 lines were similar to those seen for WT plants, possibly reflecting the segregation of edited alleles in the T_1_ generation. T_1_ plants that exhibited reduced DM symptoms were genotyped and analyzed as above. These plants included 321-5-1, 2, 5, 7, 8, 9 and 321-13-1, 2, 3, 4, 5, 9. These T_1_ plants were confirmed by PCR to be transgene-free, as were the T_0_ parental plants. PCR-sequencing and ICE analysis revealed that 7 out of 12 T_1_ *ObHSK*-edited plants, 321-5-2, 5, 7, 8 and 321-13-3, 4, 5, retained the same *ObHSK* editing patterns as their parental T_0_ plants, with Indel frequencies ranging between 20 and 22%. The remaining 5 T_1_ lines 321-5-1, 9 and 321-13-1, 2, 9 exhibited diverse editing patterns. Line 321-5-1 has 33% Indels with three distinct editing patterns (-15 nt, −18 nt, +1 nt) which result in the loss of 5 and 6 amino acids as well as other frameshifting mutations. Line 321-5-9, which has 20% Indels, retained the same editing patterns as 321-5 but with an additional 16 nt deletion that would cause a frameshifting mutation. Lines 321-13-1, 2, 9, with 19, 17, 18% Indel frequencies respectively, shared some similar editing patterns. These three lines contain −1, −10, and −13 nt deletions, all of which result in frameshifting mutations. Lines 321-13-1 and 321-13-9 also retained the same insertion patterns (+6 and +15 nt) as the 321-13 T_0_ parental plant. There was an additional editing pattern of −24 nt for lines 321-13-1 and 321-13-2, resulting in the net loss of 6 amino acids.

### Evaluation of DM Disease Phenotype on *ObHSK*-Edited Sweet Basil Mutant Plants

**Figure 4:**
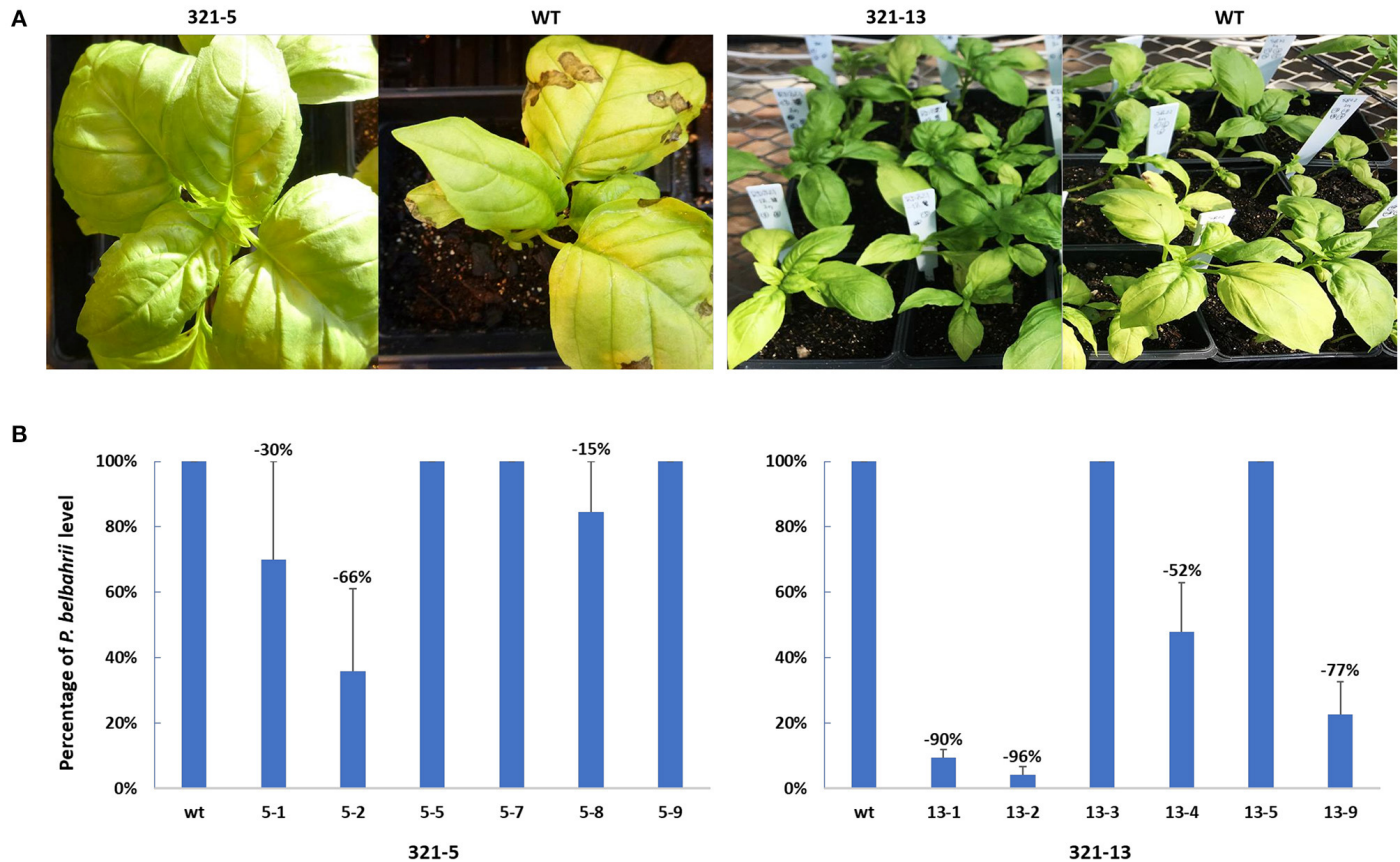
**(A)** DM resistance in gene edited plants, 321-5 and 321-13. T_1_ and WT plants were inoculated in separate batches. Disease symptoms were recorded 15 days after *P. belbahrii* inoculation. **(B)** qPCR analysis of *P. belbahrii* levels *in planta* for individual T_1_ plants, sampled in triplicate, 5 days post inoculation. *P. belbahrii* ITS2-specific primers as well as basil β-tubulin gene-specific primers were used to determine 2^−ΔΔCt^ relative levels of the pathogen. Pathogen reduction in T_1_ plants was calculated as a percentage compared to WT plants (assessed as 100% each time) and averaged from three separate qPCR assays, with standard deviations indicated.

After *ObHSK*-edited T_1_ plants were inoculated with freshly prepared and harvested *P. belbahrii* spores, the course of disease progression was assessed visually, using a previously developed index of symptoms (Wyenandt et al., 2015). Levels of pathogen DNA present inside leaves, representing colonization of host tissues, were quantitated by qPCR at 5 days post-inoculation. Figure 4A shows the phenotype of representative 321-5 and 321-13 T_1_ plants in comparison to the WT at 15 days post inoculation. The T_1_ *ObHSK*-edited plants from both lines appeared greener than the WT plants and lacked the leaf chlorosis apparent in WT plants 15 days post inoculation. 321-13 T_1_ plants performed better than did 321-5 plants with a higher proportion of 321-13 plants exhibiting healthier growth appearance than 321-5 plants. Quantitative PCR (Figure 4B) was performed on six phenotypically more resistant T_1_ *ObHSK*-edited plants from each of the 321-5 and 321-13 lines. The T_1_ plants from 321-5-1, 2, 8 lines showed 30, 66, 15% reductions of *P. belbahrii*, respectively within the inoculated leaves, compared to WT plants. In contrast, T_1_ plants from 321-5-5, 7, 9 lines contained comparable levels of *P. belbahrii* DNA as WT plants. T_1_ plants from 321-13-1, 2, 4, 9 lines showed 90, 96, 52, 77% reductions, respectively, in *in planta P. belbahrii* levels, while T_1_ plants from 321-13-3 and 321-13-5 lines had similar pathogen levels as WT plants. All 12 T_1_ plants first developed disease symptoms 25 days post inoculation while WT plants took only 10 days before the onset of disease symptoms.

## Discussion

DM is a destructive and fast spreading disease which results in yield loss and reduced quality in sweet basil. Conventional breeding for DM resistant sweet basil, a tetraploid plant, has been a challenging and time-consuming process (Pyne et al., 2015). Attempts to breed for improved DM resistance face a considerable challenge, given the ability of *P. belbahrii* to overcome resistance in extant sweet basil breeding lines. CRISPR editing represents an alternative approach which allows us to manipulate DM susceptibility genes rather than introduce classical disease resistance genes. This approach may provide more durable resistance to DM than classical breeding efforts and can also be used in concert with breeding strategies. An ortholog of *ObHSK*, Arabidopsis *AtHSK* gene has been shown to act as a susceptibility gene for the DM pathogen *Hyaloperonospora arabidopsidis* (Van Damme et al., 2005, 2009). CRISPR/Cas9 was used to disrupt the *HSK* gene from sweet basil cultivar Genoveser (Navet and Tian, 2020). However, this latter study did not report whether *HSK*-edited Genoveser sweet basil mutant plants showed any difference in susceptibility to DM. To create transgene-free *ObHSK*-edited lines in SB22 sweet basil, we employed the transient vector pRD321 to introduce mutations in *ObHSK*. Vector pRD321, expressing the *ObHSK* gRNA and Cas9 nuclease, was found to be present in 37.5% of the T_0_ lines constructed *via* biolistic transformation, in concert with a previously published report (Zhang et al., 2016). We regenerated eight mutants, five of which were transgene-free and carried various Indel mutations (Figure 3).

The mutation patterns and the Indel frequencies observed in RD321 T_0_ and T_1_ plants indicate that they are heterozygous, as expected for a polyploid plant such as sweet basil. T_0_ and T_1_ plants retained 60–70% of the WT *ObHSK* sequence in their genomes, as inferred by ICE analysis. A recent report showed that homozygous *ObHSK*-KO lines could be generated at the T_1_ generation in sweet basil cv. Genoveser (Navet and Tian, 2020) following introduction of gene editing constructs delivered by *Agrobacterium* transformation. It is possible that different vectors, delivery methods and sweet basil cultivars may contribute to differences in the efficiency and outcomes of CRISPR-gene editing. Since SB22 sweet basil is tetraploid, the *ObHSK-Apa*I gRNA target was expected to produce multiple mutation patterns in the T_0_ generation. These results revealed, respectively, that there were two types of insertion and deletion events in the T_0_ plants, with the exception of line 321-12. Both types of deletion (-1 and −13 nt) within the *ObHSK* gene led to frameshifting mutations and the presumed disruption of the functional domain of *ObHSK*. In contrast, the insertional mutations observed (+6 and +15 nt) would have added extra amino acids while preserving the reading frame and may or may not have disrupted the function of the encoded ObHSK protein.

All T_0_ plants generated in this study were phenotypically indistinguishable from WT plants, except for the sterile line 321-12, which contained seven different mutation types, including four types of frameshifting mutations and three types of mutation predicted to alter the protein coding sequence. In this line, the complement of functional *ObHSK* genes may have been more fully disrupted than in other T_0_ plants, possibly accounting for the observed growth retardation and sterility in this line. It has also been observed that the homozygous −1 frameshift T_2_ mutant Genoveser plants were dwarfs at the young seedling stage (Navet and Tian, 2020). Both results indicate that, in addition to serving as a pathogen susceptibility factor, *ObHSK* may have critical functions in plant growth and development.

Most of the T_1_ plants retained mutations inherited from their parents (Figure 3). T_1_ plants displayed more frameshifting mutations and larger polypeptide deletions (≥5 amino acids), compared to their respective parental plants. It is unclear why these T_1_ plants carried different mutation patterns compared to their parent plants, considering that PCR analysis revealed they lacked the Cas9 transgene cassette. One possibility is that the pRD321 vector was maintained episomally in somatic cells of T_0_ plants and continued to direct new gene editing events in tissues that went on to generate reproductive structures. The absence of Cas9 sequences in T_1_ plants would suggest that residual episomal plasmids in T_0_ plants were unable to pass into reproductive tissues or their products. An alternative explanation is that multiple gene edited loci underwent recombination during meiosis, resulting in novel sequences at the editing target site in the T_1_ generation (Zaman et al., 2019).

This study has shown that a number of T_1_ *ObHSK*-edited sweet basil plants were more highly resistant to DM than were WT plants. Visual assessment of *ObHSK*-edited 321-5 and 321-13 T_1_ plants showed that the resistant plants were greener in appearance compared to WT plants at 15 days post-inoculation (Figure 4 and unpublished data). This indicates that the partially edited *HSK* gene may have slowed down disease progression or attenuated pathogen virulence. In Arabidopsis, mutational disruption of *AtHSK* was associated with accumulation of homoserine, which may hamper DM infection by eliciting the plant immune system (Van Damme et al., 2009). In this study, qPCR analysis revealed a marked reduction of the pathogen load in 321-5-1, 2, 8 and 321-13-1, 2, 4, 9 T_1_ plants, particularly in lines 321-13-1 and 321-13-2, which showed up to 90 and 96% reductions, respectively. No such reduction was observed in any of the WT plants. There is no obvious correlation between the type of Indels present in *ObHSK* and the resulting level of DM resistance. Whether the six different types of *ObHSK* Indel present in 321-13-1 plants played any role in its marked (90%) pathogen reduction (Figure 4B), is unclear. In addition, despite their healthier appearance and their lack of disease symptoms, at 25 days post inoculation, lines 321-5-5, 7, 9 and 321-13-3, 5 contained similar amounts of *P. belbahrii* DNA as did WT plants at 5 days post inoculation. The disruption of *ObHSK* function caused by Indels in 321-5-5, 7, 9 and 321-13-3, 5 might have been partly compensated by non-edited alleles. One possibility is that gene editing of *ObHSK* led to the production of neomorphic or dominant-negative forms of the encoded polypeptide. In such cases, a novel phenotype, may emerge even in the presence and continued activity of WT alleles.

In this study, T_0_ and the T_1_ *ObHSK*-edited SB22 sweet basil plants were genotyped and the T_1_ plants were assayed for DM resistance. The tetraploid nature of sweet basil was reflected in the multiple Indel patterns observed within *ObHSK* gene in both T_0_ and T_1_ plants. It is not yet clear how individual gene editing events contribute to enhanced DM resistance in these sweet basil plants but it would clearly be useful to assess the effects of disrupting all alleles. The stunted and sterile phenotype displayed in the 321-12 T_0_, together with the report by (Navet and Tian, 2020) of dwarfism in homozygous *HSK*-edited sweet basil plants, suggests that *ObHSK* may have key roles to play in normal growth and development. Such plants, homozygous for disrupted *ObHSK* alleles, would not be commercially viable. However, our results indicate that even partial disruption of *ObHSK* alleles may be sufficient for a significant enhancement of DM resistance in phenotypically normal plants. Future work focusing on the molecular and genetic basis for this resistance should further facilitate strategies to create new DM resistant basils.

## Data Availability Statement

The raw data supporting the conclusions of this article will be made available by the authors, without undue reservation.

## Author Contributions

RD, JS, and ML conceived and supervised the research and revised the manuscript. XZ and YL planned and conducted research, collected and analyzed the data, and wrote the manuscript. All authors contributed to the article and approved the submitted version.

## Conflict of Interest

The authors declare that the research was conducted in the absence of any commercial or financial relationships that could be construed as a potential conflict of interest.
