# Supplementary material for: CRISPR-Editing of Sweet Basil (Ocimum basilicum L.) Homoserine Kinase Gene for Improved Downy Mildew Disease Resistance
Source: Front Genome Ed. 2021 May 12;3:629769. doi: 10.3389/fgeed.2021.629769 (PMC8525366; doi:10.3389/fgeed.2021.629769)

SUPPLEMENTAL MATERIAL for "CRISPR-editing of sweet basil (*Ocimum basilicum* L.) homoserine kinase gene for improved downy mildew disease resistance"

TABLE S1 Primers used in this study

Primers	Primer sequence
ObHSK-gDNA-F	GGTCTAGAATGGCCGCCGTCTGCCTGAAGC
ObHSK-gDNA-R	GGGAGCTCTCATCTGGGAACACTGCTGAC
RD321-F	AAGCTTCATTCGGAGTTTTTGTATC
RD321-R	GGAAATGGAAACTTCGCCGG
ObHSK-F	GGTCTAGAATGGCCGCCGTCTGCCTGAAGC
ObHSK-R	GGAAATGGAAACTTCGCCGG
ITS-qPCR-F	CGGCTGAACAGGCGCTGATTG
ITS-qPCR-R	CAACATACGACTACGGTTCATCAAG
β-tubulin-qPCR-F	CCATTCCCTCGCCTGCATTTC
β-tubulin-qPCR-R	GCTGGGATCCGCGCGATGTG

FIGURE S1 Alignment of SB22 *ObHSK* WT variants. **(A)** DNA sequences of seven different alleles were identified in SB22 *ObHSK* gene. The divergent nucleotides are boxed. The highlighted *ApaI* target sequence is conserved among all seven alleles. There are two PAM sites, AGG or TGG. Among seven alleles, wt10 is the same as *ObHSK* of Genoveser (GenBank #MT00072) and matches to our RNAseq data. **(B)** Amino acid sequences of the seven different alleles of SB22 *ObHSK* gene. The seven identified alleles encode six different ObHSK protein sequences. The divergent amino acids are highlighted by the boxes.

(A)

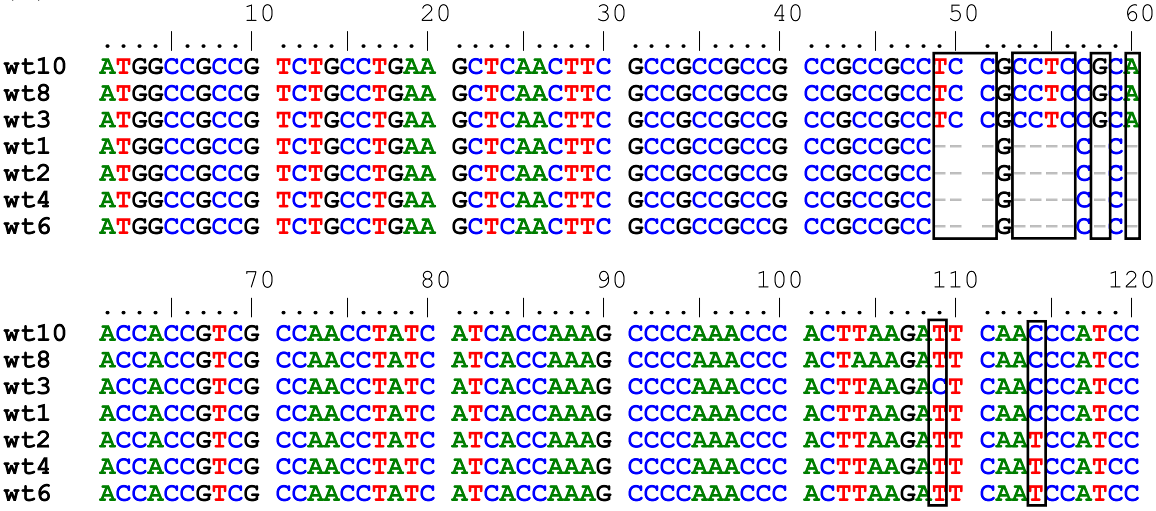

```
                 130        140        150        160        170        180
         ....|....| ....|....| ....|....| ....|....| ....|....| ....|....|
wt10     GCATCCGCAC TATCAACATC CGCTTATTCC AAATCCACTG AGCCTCTACC CGTCTTCTCC
wt8      GCATCCGCAC TATCAACATC CGCTTATTCC AAATCCACTG AGCCTCTACC CGTCTTCTCC
wt3      GCATCCGCAC TATCAACATC CGCTTATTCC AAATCCACTG AGCCTCTACC CGTCTTCTCC
wt1      GCATCTGCAC TATCAGCATC CGCCTATTCC AAATCCACTG AGCCCCTACC CGTCTTCTCA
wt2      GCATCTGTAC TATCAGCATC CGCTTATTCC AAATCCACTG AGCCCCTACC CGTCTTCTCA
wt4      GCATCTGCAC TATCAGCATC CGCTTATTCC AAATCCACTG AGCCCCTACC CGTCTCCTCA
wt6      GCATCTGCAC TATCAGCATC CGCTTATTCC AAATCCACTG AGCCCCTACC CGTCTTCTCA

                 190        200        210        220        230        240
         ....|....| ....|....| ....|....| ....|....| ....|....| ....|....|
wt10     TCCGTCAAAT CTTTCGCCCC CGCCACCGTC GCCAACTTGG GCCCTGGCTT CGACTTTCTG
wt8      TCCGTCAAAT CTTTCGCCCC CGCCACCGTC GCCAACTTGG GCCCTGGCTT CGACTTTCTG
wt3      TCCGTCAAAT CTTTCGCCCC CGCCACCGTC GCCAACTTGG GCCCTGGCTT CGACTTTCTG
wt1      TCCGTCAAAT CTTTCGCCCC CGCCACCGTC GCCAACTTGG GCCCAGGCTT CGACTTTCTG
wt2      TCCGTCAAAT CTTTCGCCCC CGCCACCGTC GCCAACTTGG GCCCAGGCTT CGACTTTCTG
wt4      TCCGTCAAAT CTTTCGCCCC CGCCACCGTC GCCAACTTGG GCCCAGGCTT CGACTTTCTG
wt6      TCCGTCAAAT CTTTCGCCCC CGCCACCGTC GCCAACTTGG GCCCAGGCTT CGACTTTCTG

                 250        260        270        280        290        300
         ....|....| ....|....| ....|....| ....|....| ....|....| ....|....|
wt10     GGATGCGCCG TAGACGGAAT CGGCGACTAC GTCAGCCTCC GAGTCGATCC AGACGTGCAC
wt8      GGATGCGCCG TAGACGGAAT CGGCGACTAC GTCAGCCTCC GAGTCGATCC AGACGTCCAC
wt3      GGATGCGCCG TAGACGGAAT CGGCGACTAC GTCAGCCTCC GAGTCGATCC AGACGTCCAC
wt1      GGATGCGCCG TTGACGGAAT CGGCGACTAC GTCAGCCTCC GAGTCGATCC GGACGTCCAC
wt2      GGATGCGCCG TTGACGGAAT CGGCGACTAC GTCAGCCTCC GAGTCGATCC AGACGTCCAC
wt4      GGATGCGCCG TTGACGGAAT CGGCGACTAC GTCAGCCTCC GAGTCGATCC AGACGTCCAC
wt6      GGATGCGCCG TTGACGGAAT CGGCGACTAC GTCAGCCTCC GAGTCGATCC AGACGTCCAC

                 310        320
         ....|....| ....|....| .
wt10     CCCGGCGAAG TTTCCATTTC C
wt8      CCCGGCGAAG TTTCCATTTC C
wt3      CCCGGCGAAG TTTCCATTTC C
wt1      CCCGGCGAAG TTTCCATTTC C
wt2      CCCGGCGAAG TTTCCATTTC C
wt4      CCCGGCGAAG TTTCCATTTC C
wt6      CCCGGCGAAG TTTCCATTTC C
```

(B)

```
                  10         20         30         40         50         60
         ....|....| ....|....| ....|....| ....|....| ....|....| ....|....|
wt10     MAAVCLKLNF AAAAAASASA TTVANLSSPK PQTHLRFNPS ASALSTSAYS KSTEPLPVFS
wt8      MAAVCLKLNF AAAAAASASA TTVANLSSPK PQTH*RFNPS ASALSTSAYS KSTEPLPVFS
wt3      MAAVCLKLNF AAAAAASASA TTVANLSSPK PQTHLRLNPS ASALSTSAYS KSTEPLPVFS
wt1      MAAVCLKLNF AAAAAA---A TTVANLSSPK PQTHLRFNPS ASALSASAYS KSTEPLPVFS
wt2      MAAVCLKLNF AAAAAA---A TTVANLSSPK PQTHLRFNPS ASVLSASAYS KSTEPLPVFS
wt4      MAAVCLKLNF AAAAAA---A TTVANLSSPK PQTHLRFNPS ASALSASAYS KSTEPLPVSS
wt6      MAAVCLKLNF AAAAAA---A TTVANLSSPK PQTHLRFNPS ASALSASAYS KSTEPLPVFS

                  70         80         90        100
         ....|....| ....|....| ....|....| ....|....| ....|..
wt10     SVKSFAPATV ANLGPGFDFL GCAVDGIGDY VSLRVDPDVH PGEVSIS
wt8      SVKSFAPATV ANLGPGFDFL GCAVDGIGDY VSLRVDPDVH PGEVSIS
wt3      SVKSFAPATV ANLGPGFDFL GCAVDGIGDY VSLRVDPDVH PGEVSIS
wt1      SVKSFAPATV ANLGPGFDFL GCAVDGIGDY VSLRVDPDVH PGEVSIS
wt2      SVKSFAPATV ANLGPGFDFL GCAVDGIGDY VSLRVDPDVH PGEVSIS
wt4      SVKSFAPATV ANLGPGFDFL GCAVDGIGDY VSLRVDPDVH PGEVSIS
wt6      SVKSFAPATV ANLGPGFDFL GCAVDGIGDY VSLRVDPDVH PGEVSIS
```

FIGURE S2 1% agarose gel electrophoresis of transgene PCR-amplification products (424 bp) from T_0 plants. gDNA was PCR-amplified with RD321-F and RD321-R primers that flank the transgene sequence between the AtU6 promoter and the 2X 35S promoter in pRD321. Lane #1-13 represent the product of: pRD321 vector, no template control for PCR reaction, WT control, 321-5, 321-10, 321-12, 321-13, 321-14, 321-4, 321-7, 321-8, WT control and 1kb+ molecular weight marker from Invitrogen.

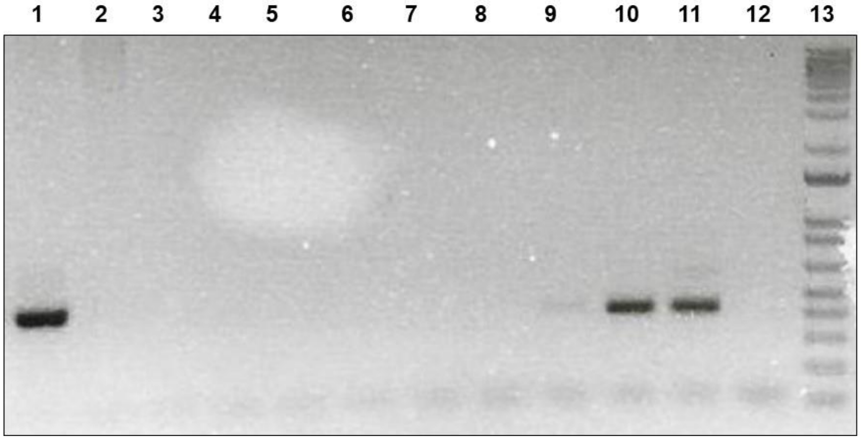

Supplement: Supplementary file 1 [file Data_Sheet_1.PDF]
